# Innovative Approaches to Avoid Antibiotic Use in Equine Semen Cryopreservation: Advancing Sustainable Reproductive Technologies

**DOI:** 10.3390/ani15101368

**Published:** 2025-05-09

**Authors:** Sonsoles Mercedes Zabala, Consuelo Serres, Natalia Montero, Francisco Crespo, Pedro Luis Lorenzo, Verónica Pérez-Aguilera, Agustín Oliet, Virginia Hijón, Santiago Moreno, Bruno González-Zorn, Luna Gutiérrez-Cepeda

**Affiliations:** 1 Animal Medicine and Surgery Department, Veterinary Faculty, Complutense University of Madrid (UCM), Avda. Puerta de Hierro s/n, 28040 Madrid, Spain; sonsoles.zabala@madrid.org (S.M.Z.); cserres@ucm.es (C.S.); fcrecas@oc.mde.es (F.C.); 2 Animal Selection and Reproduction Center, Madrid Institute for Rural, Agricultural and Food Research and Development (IMIDRA), Ctra. Colmenar Viejo a Guadalix de la Sierra, km 1, Colmenar Viejo, 28770 Madrid, Spain; agustin.oliet@madrid.org (A.O.); virginia.hijon@madrid.org (V.H.); santiago.moreno@madrid.org (S.M.); 3 Animal Health Department, Veterinary Faculty, Complutense University of Madrid (UCM), Avda. Puerta de Hierro s/n, 28040 Madrid, Spain; nmontero@vet.ucm.es (N.M.); bgzorn@ucm.es (B.G.-Z.); 4 Centro Militar de Cría Caballar de Ávila (CCFAA), C/Arsenio Gutiérrez Palacios s/n, 05005 Ávila, Spain; veronicaperezaguilera@gmail.com; 5 Physiology Department, Veterinary Faculty, Complutense University of Madrid (UCM), Avda. Puerta de Hierro s/n, 28040 Madrid, Spain; plorenzo@ucm.es

**Keywords:** frozen equine semen, simple centrifugation, filtration, SpermFilter^®^, single-layer colloidal centrifugation, Equipure^®^, semen microbial load, antibiotic-free extender, absence of antibiotics

## Abstract

This study evaluated the impact of different processing techniques on microbial load and sperm quality in frozen–thawed equine semen to identify alternatives to reduce the preventive use of antibiotics. Semen was obtained and processed under rigorous hygiene measures from ten stallions, using four protocols: Simple Centrifugation with antibiotics (S+) and Simple Centrifugation (S−), Filtration (F−) and Single-Layer Colloidal Centrifugation (C−) in an antibiotic-free extender. Microbial load in different culture media, sperm viability and motility were assessed. Microbial load results were consistent across protocols, except in Columbia 5% Sheep Blood Agar media, where S− exhibited higher microbial load than S+ (*p* < 0.05). However, F− and C− showed similar microbial loads to S+. No significant differences were observed in progressive motility, average path velocity, straight-line velocity or wobble parameters between protocols. Total motility and viability were significantly higher in S+ compared to other treatments (*p* < 0.05). Thus, regardless of antibiotics, the proposed methods achieved results similar to the traditional antibiotic-inclusive protocol in terms of microbial load and the most relevant semen quality parameters. These findings suggest that the use of F− and C−, combined with optimized hygiene measures, offers an effective alternative to reduce the prophylactic use of antibiotics in semen extenders.

## Simple Summary

Cryopreserved equine sperm is vital for global trade and genetic preservation. However, the antimicrobials included in semen extenders for this processing may be exacerbating the development of antimicrobial resistance, which is recognized by the World Health Organization as a major global health threat. This research evaluated three different processing methods—Simple Centrifugation (with/without antibiotics), Filtration and Colloidal Centrifugation, both without antibiotics—evaluating their effect on microbial load, motility and viability in frozen–thawed equine sperm. Post-freezing microbial load analysis showed a similar reduction across all methods, with Filtration and Colloidal Centrifugation without antibiotics achieving comparable values to the antibiotic control group. The results revealed no significant differences in progressive motility, average path velocity, straight-line velocity or wobble parameters between protocols. However, total motility was lower in the antibiotic-free Filtration and Colloidal Centrifugation methods compared to Simple Centrifugation with antibiotics. Viability was significantly higher in the control group with antibiotics than in the other antibiotic-free treatments. These findings suggest that the proposed alternative processing techniques, when combined with optimized hygiene practices, could control microbial load and maintain semen quality in cryopreserved sperm without antibiotics, offering a promising strategy to reduce antimicrobial resistance. Further research is recommended to validate these results and support their broader application.

## 1. Introduction

The use of cryopreserved equine sperm is important in breeding strategies as a practical and efficient method for transporting or storing semen [1]. Not only does it allow the global trade of sperm doses without the limitations of time and distance, but it also plays a crucial role in preserving the genetic material of elite stallions [2,3], thereby ensuring its availability for future breeding programs. Besides these benefits, for years, different impediments, such as increased costs, challenges in reproductive management, stallion freezability variability or reduced fertility, have prevented the widespread commercial application of frozen–thawed equine sperm [1] and led to extensive research in this area to optimize equine sperm cryopreservation technology.

One of the common strategies to help optimize equine sperm preservation technologies has been the addition of antibiotics to semen extenders [4,5,6]. Semen is a non-sterile material as it comes into contact with the normal heterogeneous microbiota of the stallion’s external reproductive organs [7,8] and can be further contaminated during semen collection and processing methods [9].

Excessive microbial proliferation in semen samples has been associated with diminished sperm quality, impaired storage preservation, lower fertility rates, and a higher incidence of reproductive disorders in mares [8]. Therefore, the incorporation of antimicrobial agents in semen extenders is a widespread practice in the equine reproductive industry, primarily as a preventive strategy during the preparation of semen doses for artificial insemination, aiming to minimize or prevent the transmission of diseases [8]. There are two types of commercial semen extenders: one containing a lower concentration of antibiotics and the other containing a relatively high concentration, which may reduce antibacterial effectiveness or may increase bacterial resistance [10]. Although cooling temperatures slow bacterial growth and freezing temperatures stop it, pathogens can survive the cryopreservation process [11,12], and it is therefore believed that the use of extenders containing antibiotics is necessary [4].

In fact, at this moment, it is not possible to find any commercial equine sperm freezing diluent that does not include antibiotics as part of its formulation. However, it is important to mention that despite the incorporation of antibiotics in semen extenders, viable bacteria have been detected in cryopreserved sperm samples from bulls [13,14,15], boars [16,17] and stallions [12].

It is important to highlight the growing concern regarding antimicrobial resistance, which has been identified by the World Health Organization (WHO) as one of the top ten global health threats, mainly driven by the misuse and overuse of antibiotics. The economic and health consequences of this issue have been considered from a One Health perspective, promoting a reduction in antimicrobial usage and limiting their incorporation in prophylactic, non-therapeutic contexts [18].

Although antibiotic-containing semen extenders are not classified as veterinary drugs, there is increasing evidence that their use can negatively affect the mare’s microbiome following artificial insemination, the surrounding environment [19,20] and potentially even human health [21]. Moreover, it is plausible that they contribute to the development of antimicrobial resistance [21,22], reinforcing the need to explore effective alternatives in reproductive biotechnologies. The search for alternatives to antibiotic use has been investigated in many mammalian species [22,23,24,25,26,27,28], with the goal of reducing antimicrobial use and preventing their inclusion in non-therapeutic situations [18]. Addressing this problem, several authors have explored different methods as potential alternatives for controlling bacterial load in equine sperm, as well as enhancing sperm quality, and simultaneously reducing the prophylactic use of antibiotics. This is particularly relevant in the equine cryopreservation industry, given that, as previously noted, there are currently no commercially available diluents that do not contain antimicrobials. Among these, physical procedures such as microfluidics, Filtration and Single-Layer Colloidal Centrifugation have been used to reduce microbial load in fresh, cooled and frozen–thawed equine sperm samples [22,23,25,29]. Other authors have investigated the incorporation of specific molecules in semen extenders of other species, such as cationic antimicrobial peptides [27] or nanoparticles [24,26], as potential alternatives to reduce antibiotic usage.

To the best of our knowledge, this is a pioneering study aimed at comparing the impact of three different processing methods (Simple Centrifugation, Filtration, and Single-Layer Centrifugation) on both microbial load and sperm quality in equine frozen–thawed sperm samples as alternatives to eliminate the prophylactic use of antibiotics in semen extenders.

## 2. Materials and Methods

### 2.1. Animals

Ten healthy adult stallions (6–27 years old) with proven fertility from the Ávila Horse Breeding Military Center (40.66° N 4.70° W), which was managed by the Spanish Ministry of Defense, were used during the breeding season of 2024 (February and March). A diverse range of equine breeds was considered to reduce potential bias in freezing ability associated with breed variability.

During the study, animals were individually housed and kept under proper and controlled feeding conditions, ensuring optimal welfare.

This study was approved by the Ethical Committee for Animal Experimentation of the Complutense Veterinary Clinical Hospital at Complutense University of Madrid (reference number 5/2021).

### 2.2. Semen Collection and Hygiene Conditions

Semen was obtained by permitting the stallion to mount a phantom and ejaculate into a Missouri-type artificial vagina (Nasco, Fort Atkinson, WI, USA), which was warmed to 45–50 °C and lubricated with a sterile, non-spermicidal gel (Priority Care^®^, IMV Technologies, L’Aigle, France) before collection. A mare in estrus was used to provide sexual stimulation. Stallions were maintained on a routine collection schedule of three times per week throughout the breeding season.

A total of ten ejaculates, one from each stallion, were obtained. Free-gel samples were quickly transported to the laboratory and kept at 37 °C for further analysis and processing.

In order to reduce environmental contamination during semen collection and processing, a strict hygienic protocol was maintained at all times to prevent contamination [22]. The artificial vagina was prepared using sterile gloves, and the stallion’s penis was washed with warm water and dried with sterile gauzes prior to semen collection. All disposable materials were sterile, and reusable items were sterilized via autoclaving or ultraviolet light exposure. To ensure sterile conditions in the laboratory, all work was carried out within the active radius of a Bunsen burner, to promote the most aseptic environment possible.

### 2.3. Experimental Design

**Figure 1:**
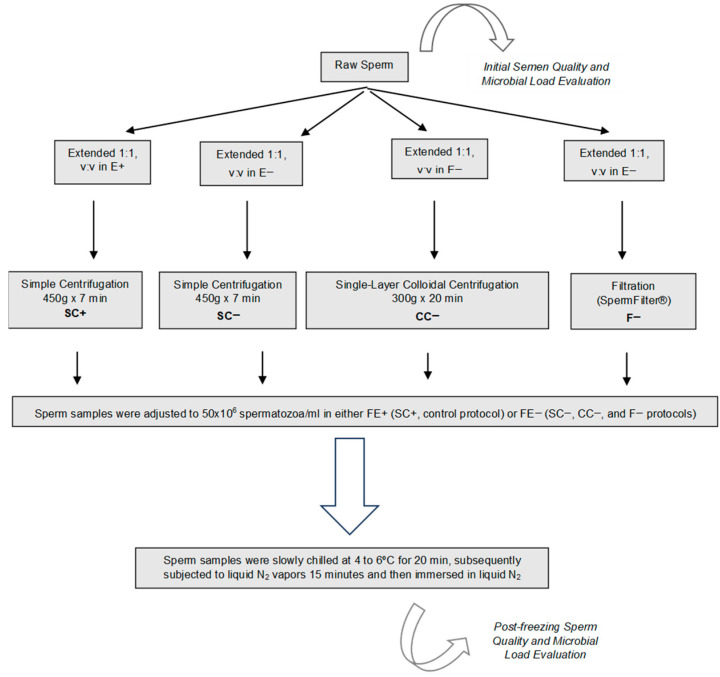
Study experimental design. E+: Equiplus^®^ extender with gentamicin. E−: Equiplus^®^ extender without antibiotics. S: Simple Centrifugation (450× *g* for 7 min) of 6 mL of extended semen with (+) and without (−) antibiotics. F−: Filtration of 6 mL of extended semen without antibiotics in the Spermfilter^®^ (Botupharma, Botucatu, Sao Paulo, Brazil). C−: Single-Layer Colloidal Centrifugation (300× *g* for 20 min) of 6 mL of extended semen without antibiotics over 6 mL of Bottom Layer Equipure^®^. After processing, each sample was diluted in freezing extender (FE) until obtaining a concentration of 50 × 10^6^ spermatozoa/mL with (FE+: BotuCrio^®^) and without antibiotic (FE−: freezing extender manufactured by us without antibiotics) to be refrigerated at 4 to 6 °C for 20 min, subsequently frozen in nitrogen vapors for 15 min and, finally, immersed directly in liquid nitrogen.

Four aliquots of each ejaculate were processed following four different protocols as described in Figure 1: Simple Centrifugation in a cooling extender with antibiotics (Equiplus^®^ with gentamicin, Minitüb, Tiefenbach, Germany) was used as the control protocol (S+: 6 mL of extended semen, 1:1; 450× *g* for 7 min); Simple Centrifugation in a cooling extender without antibiotics (Equiplus^®^ without antibiotics, Minitüb, Tiefenbach, Germany) (S−: 6 mL of extended semen, 1:1; 450× *g* for 7 min); Filtration (SpermFilter^®^) in the same cooling extender without antibiotics (F−: 6 mL of extended semen, 1:1); and Single-Layer Colloidal Centrifugation in the same cooling extender without antibiotics (C−: 6 mL of extended semen, 1:1; over 6 mL of Bottom Layer Equipure^®^, 300× *g* for 20 min).

After this initial processing, resulting samples were resuspended and adjusted to 50 × 10^6^ spermatozoa/mL in the proper volume of the freezing extender with antibiotics (BotuCrio^®^ [BioTech, Botucatu, Sao Paulo, Brazil]), in the case of the control processing protocol (S+) or in a freezing extender without antibiotics produced in our laboratory (Equiplus^®^ without antibiotics supplemented with 5% egg yolk, 4% methylformamide and 1% glycerol) for the other three processing protocols (S−, F−, and C−). After being resuspended, samples were packaged into 0.5 mL polyvinylchloride straws (IMV International, St Paul, MN, USA). Straws were then maintained at 4–6 °C for 20 min, as recommended by the manufacturer; placed in racks 4 cm above the surface of liquid nitrogen for 15 min; and finally immersed in liquid nitrogen [30].

Sperm microbial load and quality were assessed immediately in fresh semen (initial semen microbial load and quality evaluation) and after the freezing and thawing process (post-freezing sperm quality and microbial load evaluation) (Figure 1).

### 2.4. Semen Processing Techniques

Simple Centrifugation: 6 mL of extended semen (1:1, *v*:*v*) in either Equiplus^®^ with antibiotics for the Control Protocol (S+) or Equiplus^®^ without antibiotics (S−) was centrifuged (EBA 21 Centrifuge, Hettich^®^, Hong Kong, China) at 450× *g* for 7 min [31,32].

Following Simple Centrifugation, the supernatant was carefully aspirated using a sterile disposable Pasteur pipette, leaving approximately 5–10% of the seminal plasma. The recovered sperm pellets were then resuspended in the corresponding freezing extender, with antibiotics for S+ and without antibiotics for S−, to achieve a final concentration of 50 × 10^6^ spermatozoa/mL.

Filtration: 6 mL of extended semen (1:1, *v*:*v*) in Equiplus^®^ extender without antibiotics (F−) was filtered using a SpermFilter^®^ (Botucatu, Brazil), following the method described by Alvarenga et al. [25]. After Filtration, the retained spermatozoa were recovered by reversing the washing process, using the necessary volume of the corresponding antibiotic-free freezing extender to achieve a final concentration of 50 × 10^6^ spermatozoa/mL.

Single-Layer Colloidal Centrifugation: 6 mL of extended semen (1:1, *v*:*v*) in Equiplus^®^ extender without antibiotic (C−) was gently layered over 6 mL of Equipure^®^ (Nidacon, International AB, Mölndal, Sweden), pre-equilibrated at 22 °C and centrifuged (EBA 21 Centrifuge, Hettich^®^) at 300× *g* for 20 min, as described by Gutiérrez-Cepeda et al. [31]. As for Simple Centrifugation, the supernatant was aspirated, and the sperm pellets were resuspended in the freezing extender without antibiotics to achieve a final concentration of 50 × 10^6^ spermatozoa/mL.

### 2.5. Cooling and Freezing–Thawing Processing

Immediately after processing, all samples were packed into 0.5 mL straws and maintained at 4–6 °C for 20 min. Thereafter, straws were placed in racks 4 cm above the liquid nitrogen surface for 15 min and then fully submerged into liquid nitrogen [30].

Samples were maintained in liquid nitrogen for at least 48 h before evaluation. For the thawing process, straws were submerged in a water bath at 37 °C for 1 min.

### 2.6. Semen Microbial Load Evaluation

Aliquots of both raw (initial microbial load, 600 µL) and frozen–thawed (1 mL) samples were transferred into an Eppendorf tube and sent to the Department of Microbiology of the Veterinary Faculty, Complutense University of Madrid, for microbial quantification.

For analysis, 100 μL of each initial sample (dilution 0) was mixed with 900 μL of PBS solution (dilution −1). A standardized 100 μL inoculum from this dilution was then cultured in three different culture media using the spread-plate method: Columbia 5% Sheep Blood Agar (COS; Difco™, BD Diagnostics, Schwechat, Austria), Sabouraud Dextrose Agar (SDA; Difco™, BD Diagnostics, Schwechat, Austria) and Schaedler vitamin K1 5% Sheep Blood Agar (SCH; Difco™, BD Diagnostics, Schwechat, Austria). The plates were incubated at 37 °C for 24 h under aerobic conditions for COS and SDA and anaerobic conditions for SCH using a BD GasPak EZ System (BD Diagnostics). After incubation, microbial growth was evaluated, and colony-forming units (CFUs/mL) were quantified for each culture medium to assess the microbial load.

COS was used to isolate both non-fastidious and fastidious microorganisms, SDA for the culture and isolation of molds and yeasts and SCH for fastidious anaerobic bacteria.

As a quality control measure, aliquots of both cooling (E+ and E−) and freezing extenders (FE+ and FE−) were cultured following the previously described method.

### 2.7. Semen Quality Evaluation

To assess the initial semen quality, the volume of gel-free ejaculates and sperm concentration were measured in raw samples using a graduated test tube and a photometer (SMD1 Interspecies, Minitube^®^, Minitüb GmbH, Tiefenbach, Germany).

Sperm motion characteristics and vitality were evaluated both on raw sperm (initial evaluation) and frozen–thawed samples (post-thawed evaluation).

Motion characteristics were analyzed using a computer-assisted sperm motion analyzer (Sperm Class Analyzer^®^, Microptic SL, Barcelona, Spain) mounted on a microscope equipped with a heated stage and phase-contrast optics (20× objective, Optiphot-2, Nikon, Tokyo, Japan). The assessment was conducted on a 10 μL drop of extended sperm (50 × 10^6^ sperm/mL) placed on a preheated glass slide with a 22 × 22 mm coverslip. At least eight fields were examined, evaluating a minimum of 200 spermatozoa per sample. The parameters measured included percentage of total motile sperm (%; TMOT); percentage of progressively motile sperm (%; PMOT); curvilinear velocity (μm/s; VCL); straight-line velocity (μm/s; VSL); average path velocity (μm/s; VAP); linearity (%; LIN); straightness (%; STR); wobble (%, WOB); amplitude of lateral displacement (μm; ALH); and beat cross frequency (Hz; BCF). Settings for the CASA system were as follows: 60% STR threshold for progressive motility, 50% LIN threshold for circular spermatozoa, 32 frames per sequence, a minimum of 15 frames per object, a minimum object area of 25 pixels and a velocity threshold of 10 mm/s for defining immotile spermatozoa [31].

Sperm viability was assessed using the eosin–nigrosin staining technique [33]. An aliquot of semen was mixed with tempered eosin–nigrosine on a prewarmed slide, and a smear was prepared using the slide-to-slide method and then fixed with Eukitt^®^ mounting medium (ORSAtec, GmbH, Bobingen, Germany). A total of 200 spermatozoa were examined under a microscope at 40× magnification to determine the proportion of viable (unstained) and non-viable (stained) sperm cells.

### 2.8. Statistical Analysis

The normality of variable distributions was assessed using the Shapiro–Wilk test. Based on this, either parametric (one-way repeated measures ANOVA) or non-parametric (Friedman test) methods were applied for data analysis. To identify differences between treatments (S+, S−, F−, C−), post hoc tests were conducted, using either Duncan’s test or the Bonferroni correction for multiple comparisons, respectively. Results were expressed as mean, median and standard deviation. A significance level of *p* ≤ 0.05 was used to determine statistically significant differences. Data analysis was performed using SAS software (Version 9.4, SAS Institute Inc., Cary, NY, USA).

## 3. Results

### 3.1. Evaluation of Microbial Load

The initial microbial load (raw semen), expressed as colony-forming units per milliliter (CFUs/mL), for the three different culture media used is shown in Table 1.

Post-thawed microbial load showed a notable reduction compared to the initial loads across all four processing protocols. This decrease was consistently observed in both the mean ± standard deviation and median values in all culture media, demonstrating the effectiveness of the processing methods for reducing microbial contamination.

When comparing the effect among groups, no significant differences were observed between processing protocols for any of the evaluated culture media (Table 2), except for COS medium, in which samples subjected to Simple Centrifugation with antibiotics showed a significantly lower load (S+, 78 ± 203.84 CFU/mL, mean ± SD) than samples subjected to Simple Centrifugation without antibiotics (S−, 602 ± 1268.38 CFU/mL, mean ± SD).

### 3.2. Evaluation of Semen Quality

The values (mean ± SD) for initial sperm quality are presented in Table 3. Volume, concentration, TMOT, PMOT, and viability fell within the normal range for equine sperm.

When evaluating post-thawed sperm quality, no notable differences were observed between the four protocols (Table 4) in terms of the percentage of progressively motile sperm (%; PMOT), the velocity of the average path (VAP, μm/s), straight-line velocity (VSL, μm/s) and wobble (WOB, %) variables. The percentage of total motility (TMOT, %) was significantly lower in the Filtration (F-, 56.96%) and Single-Layer Colloidal Centrifugation (C−, 62.41%) protocols than in the control method (S+, 71.37%). Curvilinear velocity (VCL, μm/s) in the Single-Layer Colloidal Centrifugation group (C−, 75.96) was significantly lower than the Filtration (F−, 87.03) and Simple Centrifugation without antibiotics (S−, 87.92) protocols, but there was no significant difference from the control group (S+, 84.62). Straightness (STR, %) was significantly higher in the Filtration (F−, 70.70) and Single-Layer Colloidal Centrifugation (C−, 68.25) protocols than in the control group (S+, 63.22). Both linearity (LIN, %) and beat cross frequency (BCF, Hz) were higher in the Filtration protocol (F−, 40.91 and 18.17, respectively) than in the control group (S+, 37.02 and 14.88). And the amplitude of lateral displacement (ALH, μm) was significantly lower in the Single-Layer Colloidal Centrifugation protocol (C−, 1.81) compared to the other treatments (S+, S− and F−: 2.05, 2.11 and 1.99, respectively).

Viability (Viable %) was significantly higher in the control group (S+, 58.80) than in the other protocols (S−, C− and F−: 52.29, 42.88 and 48.86) (*p* < 0.05), with the lowest significant results for C−.

## 4. Discussion

Bacteria have been identified in frozen–thawed samples from various species [12,13,14,15,16,17] in seminal doses that included antibiotics in their composition, which may be explained by the presence of antibiotic-resistant microorganisms or the limited efficacy of the antimicrobial agents used [11,34]. In fact, some of them have been proven to carry antibiotic resistance genes [35], raising significant concerns about their potential dissemination. Given the global distribution and trade of frozen sperm, the continued use of antibiotics in extenders may contribute to the international propagation of antimicrobial resistance.

Moreover, part of the inseminated fluid is expelled from the uterus via backflow following insemination, thereby exposing the bacteria present in the vagina and surrounding environment to low concentrations of antibiotics. It has been documented that low concentrations of antibiotics are sufficient to induce antimicrobial resistance (AMR) [21].

The use of antibiotics in semen extenders may also influence the mare. The mare’s vaginal and uterine microbiota have been demonstrated to play a crucial role in fertility. A proper balance of microorganisms can help prevent infections and promote an optimal environment for embryo implantation. It is of utmost importance to emphasize the critical role played by the maintenance of a low bacterial load and a well-balanced microbiota within the female reproductive tract [19,20,36,37]. These factors are not only essential for preserving the physiological integrity of the reproductive environment but also contribute significantly to the activation and effectiveness of the uterus’s innate defense mechanisms. A stable microbial ecosystem helps prevent dysbiosis, which could otherwise compromise reproductive health and increase susceptibility to infections. Previous studies have demonstrated that the use of antibiotics in semen extenders can modify the natural microbiota of the uterus, leading to the elimination of beneficial bacteria and the proliferation of resistant strains [19]; furthermore, these changes may be associated with subclinical inflammation and reduced success rates of artificial insemination [21]. Moreover, maintaining such conditions is a key preventive measure against the development and dissemination of antimicrobial resistance, particularly in the context of repeated exposure to semen doses that may contain residual antimicrobials. As such, strategies aimed at reducing the microbial load in semen should be considered in conjunction with efforts to preserve or restore the reproductive tract’s natural microbial balance, ensuring both reproductive success and long-term biosecurity. Consequently, the utilization of antibiotic-free frozen sperm doses may emerge as a more viable and sustainable strategy that is in alignment with the mare’s reproductive physiology.

Bacteria in stallion semen have long been associated with a detrimental impact on fertility [38] as well as with a lower cryosurvival capacity [11]. Colloidal Centrifugation has been effectively used to separate spermatozoa from bacteria present in the ejaculates of humans [39], boars [40] and stallions [12,29,41,42]. Filtration has also been utilized to separate equine spermatozoa from bacteria [25]. In this study, no significant differences were observed among the four processing protocols in any of the evaluated culture media, except for Columbia 5% Sheep Blood Agar (COS), where a significant difference was found between samples processed by Simple Centrifugation with antibiotics (S+, 78 ± 203.84) and those processed by Simple Centrifugation without antibiotics (S−, 602 ± 1268.38). This may suggest that processing techniques, such as Filtration and Single-Layer Colloidal Centrifugation, both without the use of antibiotics, can achieve comparable outcomes to those obtained with the conventional Simple Centrifugation method utilizing antibiotics. Guimaraes et al. [12] found a lower bacterial load in frozen–thawed samples subjected to Colloidal Centrifugation compared to those processed by Simple Centrifugation, and Alvarenga et al. [25] found a reduction in microbial load in filtered samples compared to extended samples. Although a reduction in microbial load was achieved, none of the protocols fully eradicated all microorganisms from the semen. The use of Filtration offers practical and economic advantages as it does not require a centrifuge, making it more suitable for field applications, and it is more cost-effective than Colloidal Centrifugation.

The mean values for the assessed sperm quality variables of the ten ejaculates fell within the established reference ranges for the equine population [3]. However, when analyzing each stallion separately, we observed a diverse response to the different treatments, reflecting the natural individual variability commonly seen in semen responses in the equine population. Only those individuals meeting the commercially established standards for equine cryopreserved sperm, defined as having a progressive motility (PMOT) exceeding 50–70% in fresh semen [1,3,43] and 30–35% post-thawing [44], were included in the study.

Frozen–thawed samples exhibited lower sperm quality when compared to the initial fresh semen quality, which is in agreement with the established findings that the freezing and thawing process induces structural and functional alterations in sperm cells, negatively impacting semen quality and fertility [44]. Nevertheless, the post-thaw sperm quality parameters remained within acceptable ranges, which is consistent with expected outcomes for cryopreserved equine semen [43].

According to our results, no significant differences were observed in post-thaw quality parameters between the different processing treatments, regardless of the use of antibiotics or the incorporation of advanced stallion sperm selection techniques such as Colloidal Centrifugation or Filtration. Specifically, PMOT and certain kinematic variables (VAP, VSL and WOB) showed no statistical differences between the four processing protocols. For TMOT and viability, both the C− and F− groups showed significantly lower values than the control group (S+). For the other variables, small differences were found between treatments. Although our previous study on cooled semen [22] did not reveal significant differences in a broader range of kinetic parameters and viability, its findings are consistent with those observed in this experiment after cryopreservation, as PMOT and VAP are widely regarded as the most indicative parameters of post-thaw semen quality.

Other authors have found no significant variation between samples processed with these advanced techniques. Alvarenga et al. [25] did not find statistically significant differences in sperm motility characteristics or the percentage of membrane-intact cells, both before and after cryopreservation of equine sperm, between samples processed through Simple Centrifugation or Filtration. When considering colloidal centrifugation, other authors have not found differences in sperm quality between equine sperm samples processed by simple or Single-Layer Colloidal Centrifugation, both right after processing or freezing [12,38].

Only initial good sperm quality ejaculates were included in the experiment. It has been widely reported that selective spermatozoa techniques, specifically Colloidal Centrifugation, are mostly effective when applied to low-quality ejaculates [45,46,47]. The importance of stallion selection prior to Colloidal Centrifugation cryopreservation protocols has been described by different authors [45,46,47], and even in Filtration protocols, some authors have reported differences in post-cooling sperm quality between filtered and simple centrifuged samples only when separately assessing good and bad coolers [48].

The results of the present study show that the outcomes obtained for the main kinematic parameters using both Filtration and Single-Layer Colloidal Centrifugation techniques are similar to those obtained with Simple Centrifugation in frozen–thawed stallion semen.

Malaluang et al. (2021) demonstrated that the incorporation of antibiotics into semen processing extenders can adversely affect sperm quality, depending on the type and concentration of the antimicrobial agent [49]. Our findings support the hypothesis that stallion sperm can be successfully preserved in frozen–thawed samples without the use of antibiotics, as long as the microbial load is not too high, which can be achieved by implementing strict hygiene measures throughout the entire process of semen collection and processing [4,9,29,50]. This is particularly relevant given the growing concerns over AMR and the potential negative effects of antibiotics on sperm quality.

The use of antibiotics in stallion semen extenders has been a standard practice, as supported by European regulations, such as the Delegated Regulation (EU) 2020/686. However, growing concerns about antimicrobial resistance call for its reassessment, and in fact, other regulations, such as Regulation (EU) 2019/6, discourage its routine use and emphasize the need for alternatives to maintain hygiene in semen processing. The controversy surrounding European regulations on antibiotic use in stallion semen extenders highlights the urgent need for reassessment. With growing concerns about antimicrobial resistance, it is crucial to minimize routine antibiotic use and prioritize the development of alternative strategies to ensure proper hygiene in semen processing.

At this point, it is essential to address the translational relevance of the present findings, particularly regarding their applicability under field conditions and the practical implementation of Filtration and Colloidal Centrifugation without antibiotics. The results support the use of both methods as viable alternatives to conventional semen processing protocols, which typically rely on the inclusion of antibiotics in extenders.

Nevertheless, the adoption of these techniques in routine practice must consider the potential increase in production costs associated with their use. Although this may represent a limiting factor, particularly in large-scale operations, such additional expenses could be justified in the context of international trade involving cryopreserved semen, especially given the global imperative to reduce the prophylactic use of antimicrobials.

An additional constraint is the current lack of commercially available antibiotic-free semen extenders. The need for in-house preparation of such extenders imposes an added workload and lacks the rigorous quality control standards ensured by industrial manufacturing, potentially leading to batch-to-batch variability and inconsistent outcomes.

Although further research is necessary, these results suggest that the implementation of physical methodologies, such as Filtration and Colloidal Centrifugation or, even to a lesser extent, Simple Centrifugation, combined with improved hygiene measures, could serve as a viable strategy for the elimination of the non-therapeutic use of antibiotics in frozen semen extenders. This approach enables effective control of microbial load without compromising sperm quality, thereby contributing to a reduction in bacterial resistance risk factors within semen collection and processing facilities.

While our findings hold significant practical relevance, certain limitations should be mentioned. First, the development of antibiotic-free cryopreservation diluents is necessary, as none are currently available. Second, future research should expand the sample size and include a broader range of stallion groups to better assess the variability in semen preservation practices across the equine population. Finally, advanced microbial identification techniques (e.g., MALDI-TOF MS or the Ion 16S™ Metagenomics Workflow) should be employed to provide a more comprehensive analysis of both commensal and pathogenic bacteria, as well as their antimicrobial resistance patterns.

## 5. Conclusions

The use of Filtration and Single-Layer Colloidal Centrifugation and, to a lesser extent, Simple Centrifugation, when combined with optimized hygiene measures, offers a practical and effective alternative for reducing the prophylactic use of antibiotics in semen extenders, thereby supporting global initiatives to combat antimicrobial resistance.

## Figures and Tables

**Table 1:** Mean values of total microbial load in three different culture media in raw sperm.

Culture Media	Mean ± Std. Deviation	Minimum	Median	Maximum
COS	2436 ± 3371.24	160	700	10,400
SDA	287 ± 674.65	0	15	2170
SCH	461.11 ± 563.50	0	180	1640

Mean (mean ± SD), minimum, median and maximum values of total microbial load, expressed as colony forming units/milliliter (CFUs/mL) in raw sperm across three different culture media: Columbia 5% Sheep Blood Agar (COS), Sabouraud Dextrose Agar (SDA) and Schaedler vitamin K1 5% Sheep Blood Agar (SCH).

**Table 2:** Mean values of total microbial load in three different culture media in thawed semen samples.

Culture Media	Descriptive Statistics	S+	S−	F−	C−
COS (CFU/mL)	Mean ± SD	78 ± 203.84	602 ± 1268.38	42 ± 65.96	256 ± 478.27
	LQ	0	20	0	0
	Median	0 ^a^	135 ^b^	10 ^a,b^	35 ^a,b^
	UQ	10	290	60	160
SDA (CFU/mL)	Mean ± SD	1 ± 3.16	11 ± 14.49	19 ± 47.71	5 ± 10.80
	LQ	0	0	0	0
	Median	0 ^a^	5 ^a^	0 ^a^	0 ^a^
	UQ	0	20	0	0
SCH (CFU/mL)	Mean ± SD	6 ± 9.66	6.3 ± 12.53	8.89 ± 16.92	11 ± 28.07
	LQ	0	0	0	0
	Median	0 ^a^	0 ^a^	0 ^a^	0 ^a^
	UQ	10	10	10	10

Mean (mean ± standard deviation (SD)), Lower Quartile (LQ), median and Upper Quartile (UQ) values of total microbial load, expressed as colony forming units/mL (CFUs/mL)) in samples processed using Simple Centrifugation and extenders with antibiotics (S+), Simple Centrifugation and extenders without antibiotics (S−), Filtration and diluents without antibiotics (F−) and Single-Layer Colloidal Centrifugation and extenders without antibiotics (C−) after being frozen–thawed in three different culture media: Columbia 5% Sheep Blood Agar (COS), Sabouraud Dextrose Agar (SDA) and Schaedler vitamin K1 5% Sheep Blood Agar (SCH). Superscript letters indicate statistically significant differences (*p* < 0.05) among treatments for each variable.

**Table 3:** Average values of raw semen characteristics.

Variable	Mean ± Std. Deviation
Volume (mL)	45.52 ± 30.84
Concentration (×10^6^ sperm/mL)	212.20 ± 76.29
TMOT (%)	86.01 ± 9.86
PMOT (%)	51.94 ± 14.80
Viable (%)	70.39 ± 13.45

Average values (mean ± SD) of initial semen parameters, including volume (mL), concentration (×10^6^ spermatozoa/mL), total motile sperm percentage (%; MOT), progressively motile sperm percentage (%; PMOT) and viable sperm percentage (%; Lives) in raw semen.

**Table 4:** Mean values of frozen–thawed semen quality five minutes after thawing.

Protocol	TMOT (%)	PMOT (%)	VCL (μm/s)	VAP (μm/s)	VSL (μm/s)	STR (%)	LIN (%)	WOB (%)	ALH (μm)	BCF (Hz)	Viable (%)
S+	71.37 ^a^ ± 9.52	44.71 ^a^ ± 9.32	84.62 ^a,b^ ± 11.53	48.89 ^a^ ± 7.76	35.21 ^a^ ± 7.37	63.22 ^c^ ± 6.2	37.02 ^b^ ± 5.17	55.31 ^a^ ± 3.43	2.05 ^a^ ± 0.25	14.88 ^b^ ± 3.09	58.8 ^a^ ± 9.17
S−	64.88 ^a,b^ ± 8.96	43.63 ^a^ ± 9.06	87.92 ^a^ ± 16.25	48.79 ^a^ ± 11.92	36.85 ^a^ ± 10.51	65.97 ^b,c^ ± 5.88	37.17 ^b^ ± 5.67	53.14 ^a^ ± 4.44	2.11 ^a^ ± 0.3	16.87 ^a,b^ ± 3.59	52.29 ^b^ ± 9.41
F−	56.96 ^c^ ± 12.07	38.77 ^a^ ± 9.36	87.03 ^a^ ± 10.25	49.86 ^a^ ± 6.63	39.52 ^a^ ± 6.37	70.7 ^a^ ± 3.6	40.91 ^a^ ± 4.75	55.41 ^a^ ± 5.13	1.99 ^a^ ± 0.31	18.17 ^a^ ± 1.77	48.86 ^b^ ± 11.09
C−	62.41 ^b,c^ ± 8.28	40.2 ^a^ ± 6.12	75.96 ^b^ ± 11.72	43.24 ^a^ ± 7.52	35.44 ^a^ ± 9.14	68.25 ^a,b^ ± 3.81	39.53 ^a,b^ ± 3.3	55.24 ^a^ ± 2.84	1.81 ^b^ ± 0.23	15.54 ^b^ ± 2.26	42.88 ^c^ ± 9.77

Average values (expressed as mean ± standard deviation) for percentage of total motile semen (TMOT, %), percentage of progressive motile semen (PMOT, %), curvilinear velocity (VCL, μm/s); average path velocity (VAP, μm/s), straight-line velocity (VSL, μm/s), straightness (STR, %), linearity (LIN, %), wobble (WOB, %), amplitude of lateral displacement (ALH, μm), beat cross frequency (BCF, Hz), and viability (Viable %) after five minutes of incubation of frozen–thawed semen across four processing protocols: Simple Centrifugation with antibiotics, S+; Simple Centrifugation without antibiotics, S−; Filtration without antibiotics, F−; and Single-Layer Colloidal Centrifugation without antibiotics, C−. Superscript letters indicate significant differences between treatments (*p* < 0.05) for TMOT, VCL, STR, LIN, ALH, BCF and Lives.

## Data Availability

The data from this study can be obtained upon request from the corresponding author. They are not publicly accessible due to privacy limitations.

## Abbreviations

The following abbreviations are used in this manuscript:

WHO	World Health Organization
S+	Simple Centrifugation with antibiotics
S−	Simple Centrifugation without antibiotics
F−	Filtration without antibiotics
C−	Single-Layer Colloidal Centrifugation without antibiotics
EU	European Union
AMR	Antimicrobial resistance
CFUs	Colony-forming units
E+	Equiplus^®^ extender with gentamicin
E−	Equiplus^®^ extender without antibiotics
FE+	Freezing extender with antibiotics
FE−	Freezing extender without antibiotics
COS	Columbia 5% Sheep Blood Agar
SDA	Sabouraud Dextrose Agar
SCH	Schaedler vitamin K1 5% Sheep Blood Agar
SD	Standard deviation
LQ	Lower Quartile
UQ	Upper Quartile
TMOT	Total motility
PMOT	Progressive motility
VCL	Curvilinear velocity
VAP	Average path velocity
VSL	Straight-line velocity
STR	Straightness
LIN	Linearity
WOB	Wobble
AHL	Amplitude of lateral displacement
BCF	Beat cross frequency
